# Pigment epithelial detachment composition indices (PEDCI) in neovascular age-related macular degeneration

**DOI:** 10.1038/s41598-022-27078-x

**Published:** 2023-01-02

**Authors:** Amrish Selvam, Sumit Randhir Singh, Supriya Arora, Manan Patel, Arnim Kuchhal, Stavan Shah, Joshua Ong, Mohammed Abdul Rasheed, Shanmukh Reddy Manne, Mohammed Nasar Ibrahim, José-Alain Sahel, Kiran Kumar Vupparaboina, Jay Chhablani

**Affiliations:** 1 Department of Ophthalmology, University of Pittsburgh, Pittsburgh, PA USA; 2 Nilima Sinha Medical College and Hospital, Rampur, India; 3 Bahamas Vision Center and Princess Margaret Hospital, Nassau, NP Bahamas; 4 Fox Chapel High School, Pittsburgh, PA USA; 5 School of Optometry and Vision Sciences, University of Waterloo, Waterloo, ON Canada; 6 BJ Medical College, Ahmedabad, Gujarat India; 7 Department of Electrical Engineering, Indian Institute of Technology Hyderabad, Hyderabad, Telangana India

**Keywords:** Biomarkers, Medical research, Computational biology and bioinformatics, Image processing

## Abstract

We provide an automated analysis of the pigment epithelial detachments (PEDs) in neovascular age-related macular degeneration (nAMD) and estimate areas of serous, neovascular, and fibrous tissues within PEDs. A retrospective analysis of high-definition spectral-domain OCT B-scans from 43 eyes of 37 patients with nAMD with presence of fibrovascular PED was done. PEDs were manually segmented and then filtered using 2D kernels to classify pixels within the PED as serous, neovascular, or fibrous. A set of PED composition indices were calculated on a per-image basis using relative PED area of serous (PEDCI-S), neovascular (PEDCI-N), and fibrous (PEDCI-F) tissue. Accuracy of segmentation and classification within the PED were graded in masked fashion. Mean overall intra-observer repeatability and inter-observer reproducibility were 0.86 ± 0.07 and 0.86 ± 0.03 respectively using intraclass correlations. The mean graded scores were 96.99 ± 8.18, 92.12 ± 7.97, 91.48 ± 8.93, and 92.29 ± 8.97 for segmentation, serous, neovascular, and fibrous respectively. Mean (range) PEDCI-S, PEDCI-N, and PEDCI-F were 0.253 (0–0.952), 0.554 (0–1), and 0.193 (0–0.693). A kernel-based image processing approach demonstrates potential for approximating PED composition. Evaluating follow up changes during nAMD treatment with respect to PEDCI would be useful for further clinical applications.

## Introduction

Age-related macular degeneration (AMD) is a leading cause of blindness in the developed world, primarily affecting individuals over the age of 60 years and accounting for 8.7% of all the blindness worldwide^1,2^. Wet AMD, also referred to as exudative or neovascular AMD (nAMD), is the less common type. It accounts for only 10–15% of cases but is responsible for the majority of severe central vision loss in patients with AMD^3,4^. Choroidal neovascularization (CNV) is the hallmark feature of nAMD, which can lead to rapidly progressive vision loss^5^. In CNV, abnormal blood vessels grow from the choroidal vasculature leading to disruption of the Bruch’s membrane. These vessels can also leak blood and filtrate into the retina leading to fluid in the intraretinal, subretinal, and sub-retinal pigment epithelial (RPE) spaces^6,7^.

Elevation of the RPE layer, referred to as a pigment epithelial detachment (PED), is a frequent finding seen in patients with nAMD and usually indicates the presence of CNV^4^. Different types of PEDs exist depending on the type of material found within them. To name a few, PEDs may be filled with drusenoid material, serous fluid, neovascular tissue, and/or fibrous tissue. The size and type of PED can greatly influence disease progression^8–10^. For example, PEDs with fibrous and neovascular tissue are a risk factor for RPE rips and tears with an incidence around 10%^11^. The presence of PEDs is prognostic of poor visual outcomes and thus are closely evaluated by clinicians^4,12,13^.

Patients with nAMD undergo routine optical coherence tomography (OCT) imaging to help guide treatment and monitor disease progression^1,3^. OCTs provide a non-invasive, non-contact method of visualizing cross-sections of the retinal and choroidal structures. OCT scans can therefore provide clear views of abnormal fluid and tissues within these structures^10^. PEDs can be identified by hyporeflective, hyperreflective, and/or irregularly reflective pixels beneath the RPE. PEDs with drusenoid material are usually uniform, hyperreflective, and rounded in shape^4^. PEDs with serous fluid are usually uniform and hyporeflective with a smooth contour. PEDs with neovascular and/or fibrous tissue, also termed fibrovascular PED, have a more varied appearance. They can have an irregular shape with varying reflectivity^4^.

The term fibrovascular refers to neovascular infiltration associated with fibrotic tissue, and therefore is an encompassing term that captures both neovascular and fibrous tissue^5^. This commonly used term is a result of the difficulty associated with distinguishing between neovascular tissue and fibrous tissue. On fundus image, the two tissues often overlap spatially making it nearly impossible for clinicians to differentiate. However, with the advent of OCT, and particularly high-definition OCT imaging, PEDs can be viewed in greater detail. Yet, similar raw intensity values between the tissue types and noise within the image make the task difficult to perform with the naked eye.

In this study, we use an automated method for analyzing the OCT-based reflectivity to quantify presumed components of PEDs. We hypothesize that there are inherent differences between serous, neovascular, and fibrous material within PEDs on OCTs that can be revealed by using image processing techniques. Our objective is to use a series of image processing techniques to approximate these components and estimate the relative areas of each tissue type within PEDs in patients with nAMD.

## Methods

This was a retrospective cross-sectional study involving patients with nAMD who attended the University of Pittsburgh Medical Center Eye and Ear Institute. The study was carried out in accordance with the Declaration of Helsinki and approved by the institutional review board (IRB) from the University of Pittsburgh. Written informed consent was obtained from all study participants.

All subjects received a complete history and comprehensive ophthalmological exam including best-corrected visual acuity (BCVA), intraocular pressure measurement, slit-lamp microscopy, and dilated fundus exam to confirm nAMD and rule out other ocular pathologies. Inclusion criteria for this study included patients with confirmed nAMD with at least one PED captured by B-scan. The minimum height and width of evaluated PEDs were 100 microns. Exclusion criteria included complicated nAMD, drusenoid PED, and the presence of other ocular diseases besides nAMD. Complicated nAMDs were defined as cases where RPE rips were present. Drusenoid PEDs were defined by clinical evaluation and excluded to narrow the focus of the study to nAMD. Eyes that received prior anti-vascular endothelial growth factor (anti-VEGF) treatment and eyes that were treatment naïve were both included.

Spectral Domain Optical Coherence Tomography (SD-OCT) images were acquired using the Heidelberg Spectralis Device (Heidelberg Engineering, Heidelberg, Germany) for all patients. Single scan passing through the fovea was used for image analysis. Each B-scan had a resolution of 1536 × 496 pixels with an intensity range of 0–255.

### Image pre-processing

To ensure accurate analysis of OCT images, a series of pre-processing steps were applied: (1) linear normalization and (2) shadow compensation^14^. The acquired scans displayed varied intensity distributions. To account for these differences, all B-scans were standardized using linear normalization to a range of 0–1. Since the area of interest lies below the RPE layer, another concern was signal extinction, visualized as shadows, caused by fluid and vessels in the retina^15,16^. To correct for this signal extinction, shadow compensation was applied to all scans. This method corrected for shadows by calculating a unique compensation factor on a per-pixel basis by evaluating idiosyncrasies in the OCT^16^. Scans adjusted by pre-processing steps are referred to as original processed images (*I*_0_).

### Segmentation of PED

Subsequently, PEDs from each scan were manually segmented by a trained annotator and verified by an expert clinician. Segmentations delineated the outer boundary of the entire PED and were performed using the ImageJ software v1.53m. The polygon selector tool was used to outline the suspected PED, and the “Fit Spline” feature was used to smooth the segmentation.

### PED analysis

**Figure 1:**
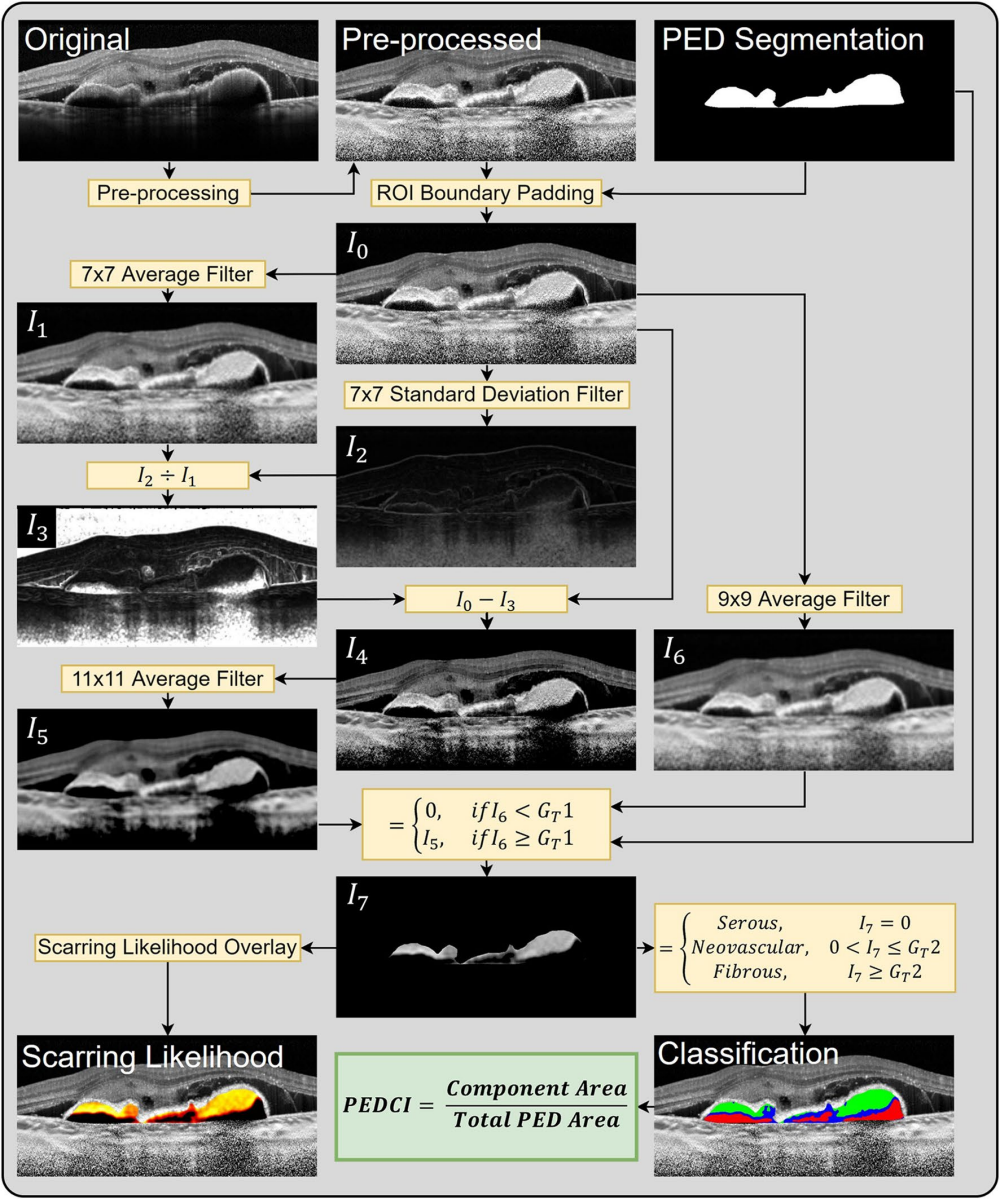
Proposed methodology for classification of serous, neovascular, and fibrous tissues in pigment epithelial detachments (PED) and derivation of PED composition indices (PEDCI). Starting with the original B-scan image in the top left, pre-processing steps including linear normalization and shadow compensation were applied. The segmented PED was subsequently used to pad the region of interest in the processed image, *I*_0_. The transformation between *I*_0_ to *I*_7_ involves a series of kernel-based filtering steps. Classification of components within the PED was performed using *I*_7_. PEDCI-S was derived from the ratio of the serous component area to total PED area, PEDCI-N was derived from the ratio of the neovascular component area to total PED area, and PEDCI-F was derived from the ratio of the fibrous component area to total PED area.

To characterize the fluid and tissue within a PED, a method of delineating serous, neovascular, and fibrous tissue was desired. To perform this segmentation step, multiple image processing techniques were explored including adaptive thresholding using Otsu’s method and unsupervised learning via a K-means based algorithm. While Otsu’s method and similar adaptive thresholding techniques have shown promise in deriving other OCT biomarkers such as choroidal vascularity index, such techniques yielded suboptimal results for PED characterization^15,17^. Therefore, a custom pixel-wise scarring likelihood metric was computed for each pixel within the PED using multiple filtering steps. This metric, ranging from 0 to 1, was designed to estimate a given pixel’s likelihood of belonging to either serous fluid, neovascular tissue, or fibrous tissue. Neovascular tissue is defined as having a predominately vascular component, while fibrous tissue is defined as having a predominately extracellular matrix component. A score of zero corresponds to serous fluid, a low-intermediate score (0–0.6) corresponds to neovascular tissue, and a high score (0.6–1) corresponds to fibrous tissue. To quantify the composition of material within the PED, a collection of PED composition indices (PEDCI) was derived by calculating the relative area of serous (PEDCI-S), neovascular (PEDCI-N), and fibrous (PEDCI-F) material. To calculate the pixel-wise scarring likelihood and PEDCI scores, a series of filters were applied to generate modified images. Prior to these filtering steps, the PED region was padded using symmetric reflection at the boundary. A schematic of the overall methodology is shown in Fig. 1.

The filtering steps used were as follows. A 7 × 7 averaging kernel was applied to the original processed image, *I*_0_, generating *I*_1_. A 7 × 7 standard deviation filter was applied to *I*_0_, generating *I*_2_. *I*_2_ was divided by *I*_1_ on a per pixel basis generating *I*_3_. *I*_3_ was subtracted from *I*_0_, generating *I*_4_. A 11 × 11 averaging filter was applied to *I*_4_, generating *I*_5_. A 9 × 9 averaging filter was applied to *I*_0_, generating *I*_6_. *I*_7_ was computed within the PED segmentation by comparing values in *I*_6_ against a global threshold (*G*_*T*_1). If the value of the pixel in *I*_6_ was less than *G*_*T*_1, the corresponding pixel in *I*_7_ was set to 0, otherwise the pixel in *I*_7_ was set to the corresponding pixel in *I*_5_. The intensity of pixels of *I*_7_ reflects the pixel-wise scarring likelihood. The 7 × 7 filters were used to derive the local heterogeneity of the image while the larger 9 × 9 and 11 × 11 filters were used near the end of the algorithm when aggregation of pixels was desired.

Each pixel was subsequently categorized as serous, neovascular, or fibrous using *I*_7_. A value of 0 was categorized as serous fluid. To categorize non-serous pixels, a global threshold (*G*_*T*_2) was used. Scores greater than *G*_*T*_2 were categorized as fibrous, and scores lower than *G*_*T*_2 were categorized as neovascular. Initially, *G*_*T*_1 and *G*_*T*_2 were arbitrarily set, but repeated trials were reviewed by an expert clinician to determine final thresholds. On final analysis, *G*_*T*_1 and *G*_*T*_2 were held at constant values for all images, set independently of evaluators. These filtering steps are summarized in Eqs. (1) and (2). Finally, PEDCI was determined using the calculations represented in Eqs. (3–5). Pre-processing and image analysis steps were performed using Matlab 2020b.

1 $$ I_{7} = \left\{ \begin{array}{ll} {0,} & \quad if\quad AVG\left( {I_{0} , 9} \right) < G_{T} 1 \\ AVG\left[ {I_{0} - \left( \begin{array}{c} \frac{STD\left( {I_{0} ,\;7} \right)}{AVG\left( {I_{0} ,\;7} \right)} \\ \end{array} \right), 11} \right], &\quad if\quad AVG\left( {I_{0} , 9} \right) \ge G_{T} 1 \\ \end{array} \right.$$

2 $$Pixel\,Classification = \left\{ {\begin{array}{ll} {Serous,} \hfill & {\quad I_{7} = 0} \hfill \\ {Neovascular,} \hfill & {\quad 0 < I_{7} \le G_{T} 2} \hfill \\ {Fibrous,} \hfill & {\quad I_{7} \ge G_{T} 2} \hfill \\ \end{array} } \right.$$

3 $${\textit{PEDCI-S}} = \frac{Serous\,Area}{{Total\,PED\,Area}}$$

4 $${\textit{PEDCI-N}} = \frac{Neovascular\,Area}{{Total\,PED\,Area}}$$

5 $${\textit{PEDCI-F}} = \frac{Fibrous\,Area}{{Total\,PED\,Area}}$$

The image processing algorithm, including the tuning of global threshold values and filter operations, were determined using a training dataset of 15 eyes from 15 patients with neovascular age-related macular degeneration. These eyes were independent from the validation dataset and were excluded from evaluation.

### Evaluation strategy

Relative and absolute areas of each fluid and tissue type were calculated within the PED. These regions were then overlaid on the original processed image for visualization. While ground truth classification of the material within the PED is important for evaluation of algorithm accuracy, delineating between the different tissue types using the naked eye alone is challenging. Therefore, we evaluated the accuracy of the algorithm using masked observer grading. Two expert clinicians were tasked with grading the accuracy of the segmentation and classification within the PED in a masked fashion. If a certain fluid or tissue type was absent, no grade was given. Each grader evaluated each PED at two different timepoints taken at least 24 h apart and were masked to their own grades as well as other observer’s grades. Images were presented in a randomized order. Intraclass correlations (ICC) were used to evaluate for intra-observer repeatability (between the same grader) and inter-observer reproducibility (between different graders). Scores were reported on a scale of 0–100, with 0 being inaccurate and 100 being accurate. Graders were masked to their own grades and to the grades of other graders. Statistical analysis was performed using Stata16 and ExcelStats.

## Results

A total 43 eyes from 37 patients (23 female, 14 male) with nAMD were included for validation of the proposed methodology in the study. The mean age of the validation cohort was 75.2 ± 14.4 years. Serous fluid, neovascular tissue, and fibrous tissue were identified in 26, 38, and 24 of the 43 eyes respectively.

**Figure 2:**
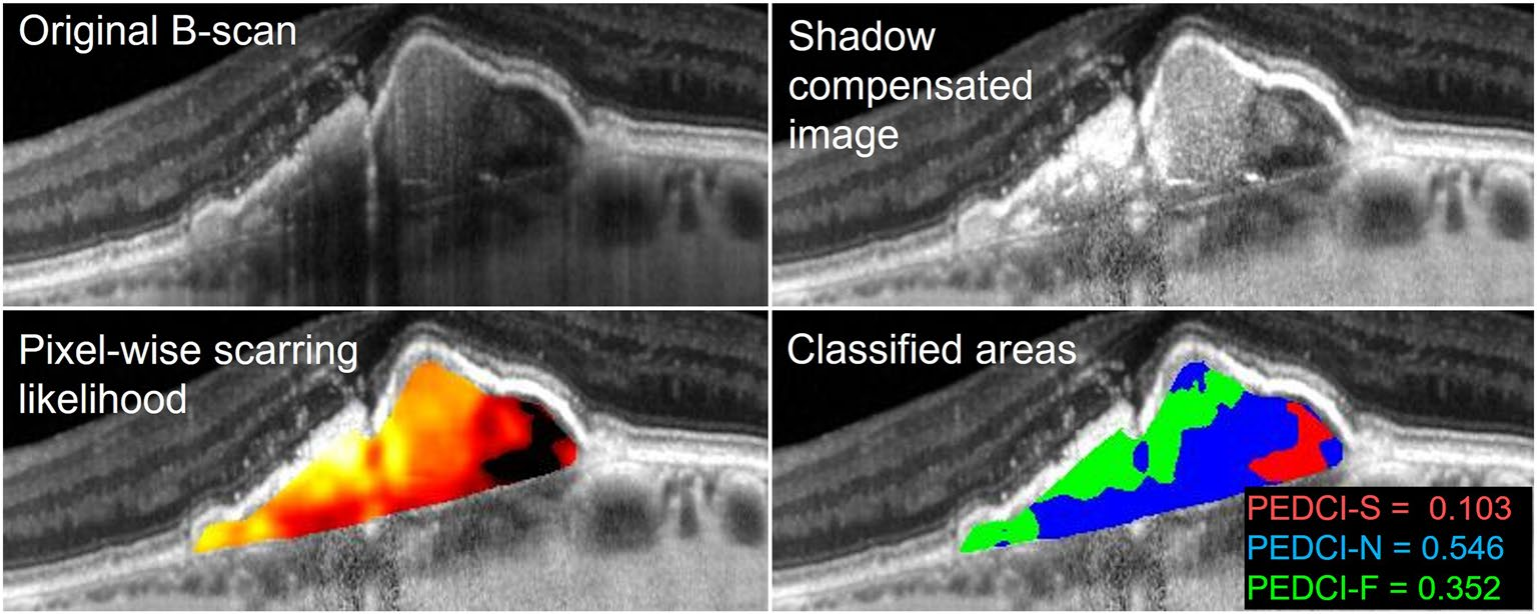
Classification of pixels within a pigment epithelial detachment (PED) of a patient with neovascular age-related macular degeneration. The original B-scan was taken using spectral domain optical coherence tomography. Results from subsequent shadow compensation and computed per-pixel scarring likelihood are shown. Classified areas include serous (red), neovascular (blue), and fibrous (green) components of the PED. Classified areas were used to compute the PED composition indices: serous (PEDCI-S), neovascular (PEDCI-N), and fibrous (PEDCI-F).

**Figure 3:**
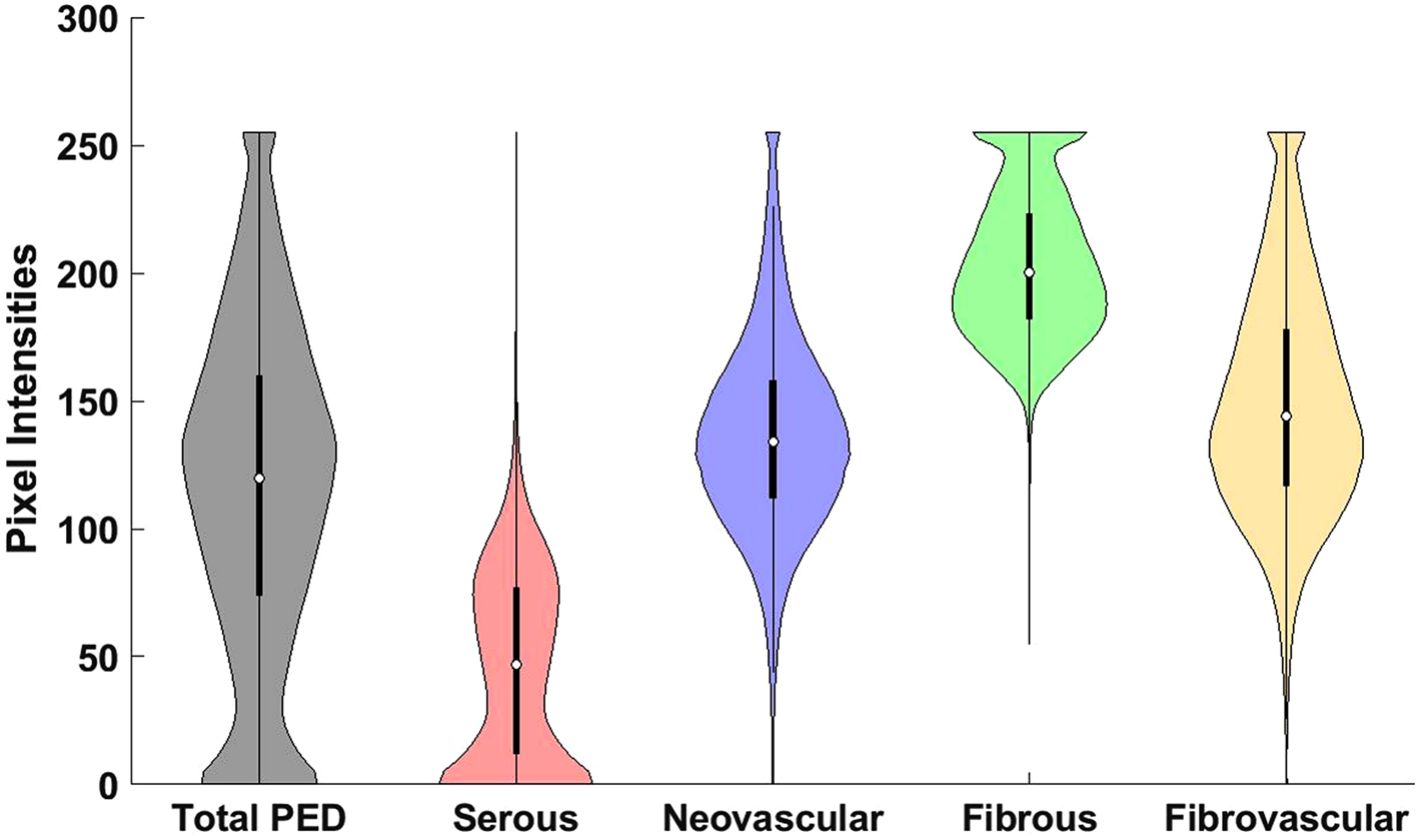
Violin plot showing the distribution of pixel intensities from different components of a pigment epithelial detachment (PED). Pixel intensities correspond to pre-processed spectral-domain optical coherence tomography images taken from 43 eyes with neovascular age-related macular degeneration. Mutually exclusive classified components include serous, neovascular, and fibrous. Fibrovascular is the combined distribution of neovascular and fibrous tissue. Total PED is the combined distribution of serous, neovascular, and fibrous tissue. The overlap in the distributions indicates the difficulty in separating tissue types, particularly neovascular and fibrous tissue. The skewed distribution of fibrovascular tissue indicates more than one underlying tissue type.

Mean total PED area was 0.28 ± 0.41 mm^2^. When present, mean absolute serous, neovascular, and fibrous areas in mm^2^ were 0.16 ± 0.36, 0.15 ± 0.20, and 0.06 ± 0.06 respectively. When present, mean relative serous, neovascular, and fibrous areas were 41.7% ± 29.8%, 58.3% ± 28.2%, and 21.1% ± 21.3% respectively. The discrepancy of serous fluid and neovascular tissue areas and percentages can be attributed to relative PED size. This is reflected by the median absolute serous, neovascular, and fibrous areas in mm^2^, which were 0.06, 0.09, and 0.04 respectively. The mean PEDCI-S score was 0.253 ± 0.309, with a range of 0 to 0.952. The mean PEDCI-N score was 0.554 ± 0.267, with a range of 0 to 1. The mean PEDCI-F score was 0.193 ± 0.209, with a range of 0 to 0.693. An example of the classified PED from one of the patients is shown in Fig. 2. Distribution of image intensities across all patients are shown for each PED component in Fig. 3.

The mean graded score of the segmentation of the PED was 96.99 ± 8.18. The mean graded scores of the classification of serous, neovascular, and fibrous tissue were 92.12 ± 7.97, 91.48 ± 8.93, and 92.29 ± 8.97 respectively as graded by two independent clinicians.

**Figure 4:**
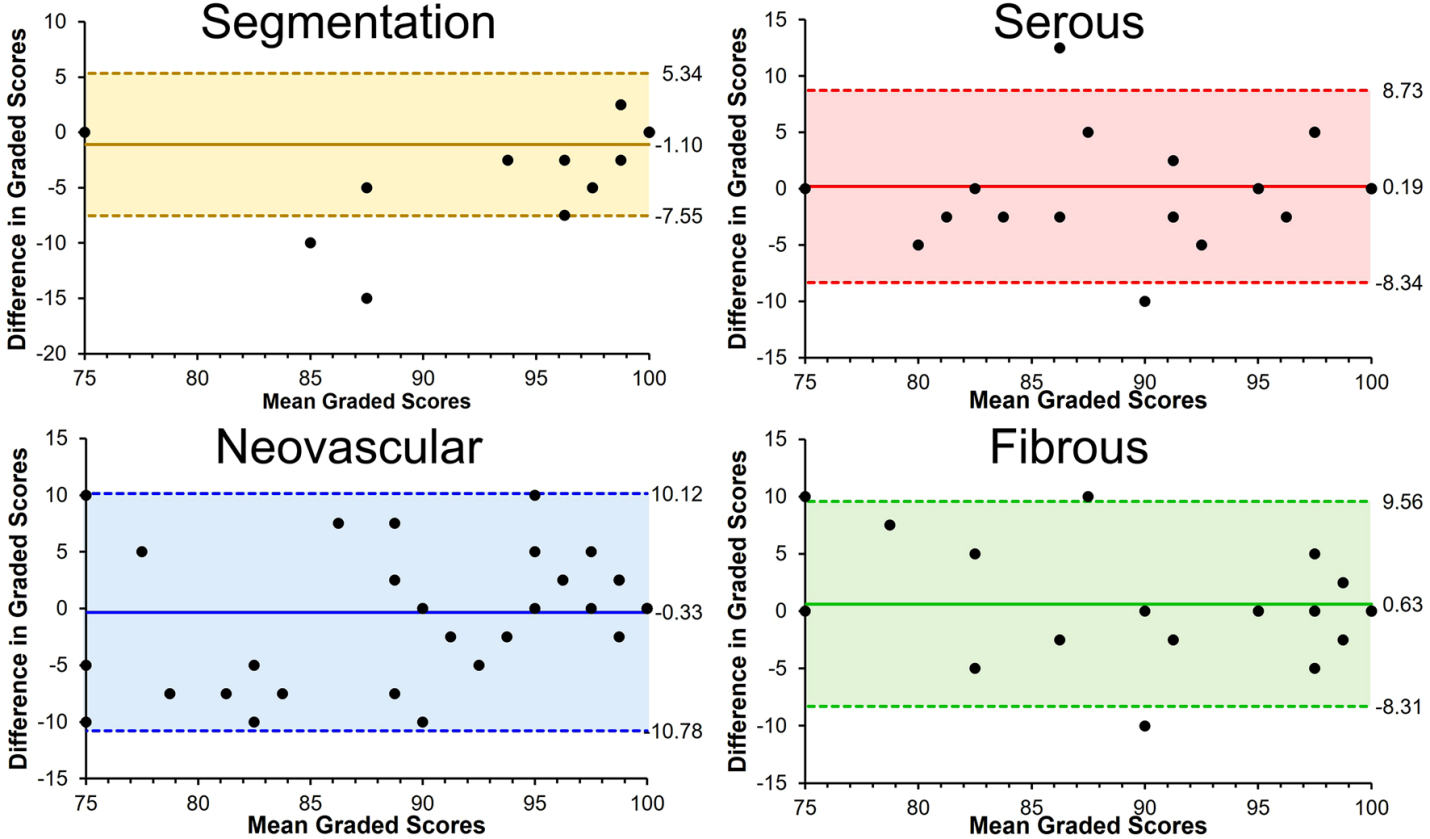
Bland–Altman plots capturing the difference in graded scores between two expert clinicians in evaluating accuracy of segmentation and classification of serous, neovascular, and fibrous components of pigment epithelial detachments (PED). Shaded areas capture two standard deviations from the mean. Significant outliers in segmentation scores demonstrated disagreement of neovascular vs non-neovascular PED.

The ICCs for repeatability for grader 1 were 0.98, 0.86, 0.88, and 0.84; for grader 2 were 0.95, 0.76, 0.79, and 0.82 for accuracy of segmentation and classification of serous, neovascular, and fibrous tissue respectively. The mean ICCs for repeatability was 0.86 ± 0.07. The ICC for reproducibility were 0.89, 0.84, 0.82, and 0.87 for accuracy of segmentation and classification of serous, neovascular, and fibrous tissue respectively. The mean ICCs for reproducibility was 0.86 ± 0.03. Bland–Altman plots for ICCs for reproducibility are shown in Fig. 4.

**Figure 5:**
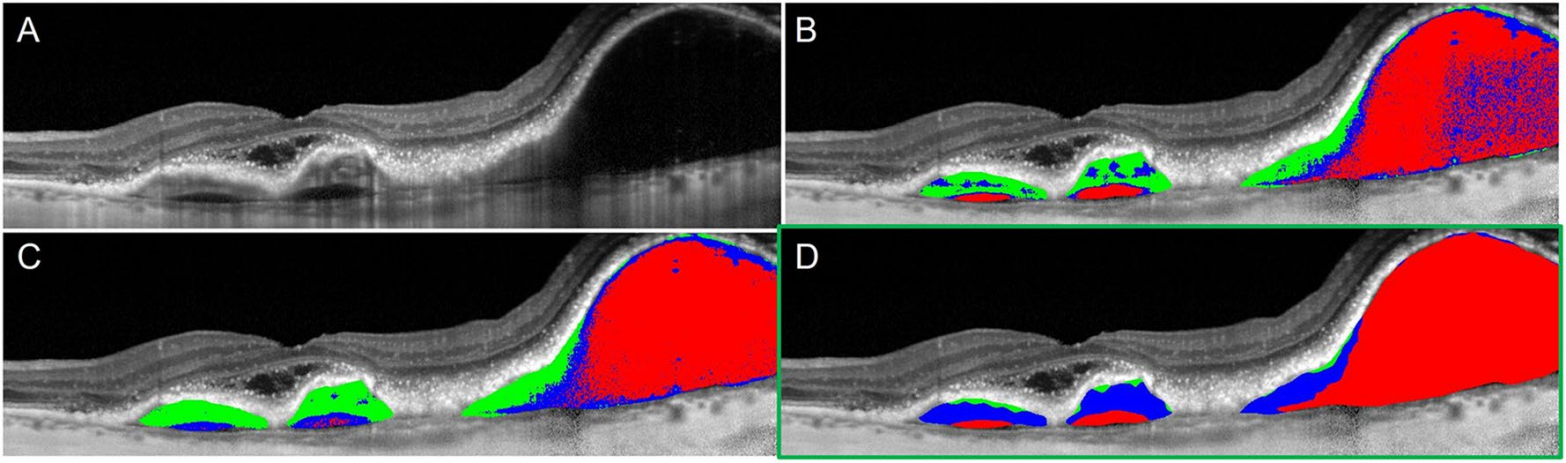
Classified components of a pigment epithelial detachment (PED) using different image techniques on an optical coherence tomography B-scan taken from a patient with neovascular age-related macular degeneration. Classified areas of the PED include serous (red), neovascular (blue), and fibrous (green) components. Original b-scan (**A**). Adaptive thresholding using Otsu’s method (**B**). Unsupervised learning via K-means clustering (**C**). Final pigment epithelial detachment composition index technique using kernel-based image processing techniques (**D**).

Results from explored image processing techniques are shown in Fig. 5. In addition to the described methodology, segmentation results from adaptive thresholding and unsupervised machine learning are displayed. Qualitatively, these techniques displayed increased heterogeneity in segmented components and were more sensitive to noise, which led to greater misclassification of pixels. Unsupervised learning methods were prone to underestimation of serous components. Delineation of the fibrous tissue cannot be clearly seen in either of these techniques.

### Preliminary analysis of clinical correlation

**Figure 6:**
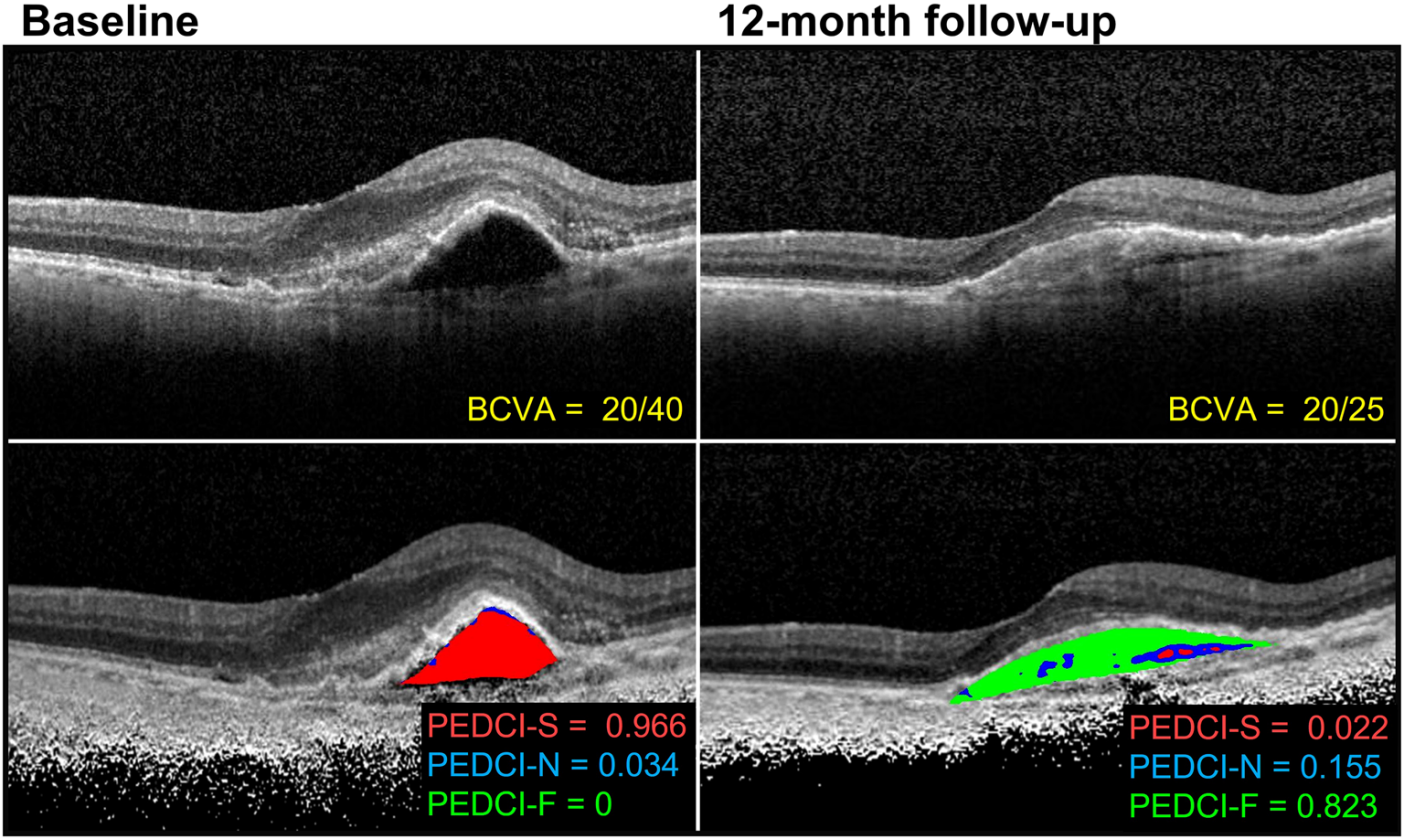
Optical coherence tomography images from a patient with neovascular age-related macular degeneration at baseline and 12-month follow-up. Baseline images are treatment naïve. The patient received six intravitreal aflibercept injections before the 12-month visit. Best-corrected visual acuities (BCVA) are reported along with calculated PED composition indices: serous (PEDCI-S), neovascular (PEDCI-N), and fibrous (PEDCI-F). Classified areas include serous (red), neovascular (blue), and fibrous (green) components of the PED.

While the aim of this study is to provide an automated method of quantifying PEDs via the PEDCI biomarkers, we suspect that such biomarkers, particularly PEDCI-F which infers presumed scarring, will be used by clinicians to assess for treatment response in patients with nAMD. To explore this clinical application for follow-up evaluation, we have included a testing dataset of SD-OCT B-scans from a patient with nAMD. B-scans were taken at a baseline treatment-naïve visit and 12-month follow-up visit following anti-vascular endothelial growth factor (anti-VEGF) treatment. OCT images along with best-corrected visual acuity and PEDCI analysis are shown in Fig. 6.

The patient received six intravitreal aflibercept injections by the 12-month visit. An improvement in BCVA was observed at the follow-up visit from 20/40 at baseline to 20/25 at the 12-month follow-up. PEDCI-F increased from 0 at baseline to 0.823 at follow-up and PEDCI-S decreased from 0.966 to 0.022. The patient had a primarily serous composition at baseline followed by a primarily fibrous composition at follow-up.

## Discussion

Our study provides an automated method for analyzing PEDs within high resolution SD-OCT B-scans in patients with nAMD. We found that a kernel-based approach can discriminate between serous, neovascular, and fibrous tissue within a PED, with a mean ICC for repeatability and reproducibility of 0.86 ± 0.07 and 0.86 ± 0.03 respectively. To quantify the PED components, PEDCI biomarkers were calculated. Since these biomarkers were calculated as a ratio of individual components to total PED area, the repeatability and reproducibility of classification accuracies serves as a good proxy for the evaluation of PEDCI.

To determine the methodology of our image processing approach, we started with a general schema of known OCT signatures. Serous fluid is generally hyporeflective and uniform on SD-OCT^4,10^. Therefore, to determine if a pixel was representative of serous fluid, a global threshold was applied along with an averaging filter to limit the effects of erroneous pixels caused by image acquisition noise. Distinguishing between neovascular and fibrous tissue was a more challenging task due to their overlapping raw pixel intensity distribution (Fig. 3). Inclusion of drusen, which have distinct clinical features from fibrovascular lesions but similar pixel intensity distributions, would complicate a pixel-wise separation approach further. This is an additional reason why this study was limited to non-drusenoid PEDs in nAMD. When analyzing fibrovascular lesions, both neovascular and fibrous tissues are found in PEDs with a generally irregular appearance and neighboring SRF, which makes clear delineation difficult^13^. However, given enough resolution within a B-scan, neovascular tissue should have more heterogeneity in its reflectiveness. Within neovascular tissue, hyperreflective areas of supporting tissues should be speckled by hyporeflective pockets of fluid within the abnormal small blood vessels. In contrast, fibrous tissue should be uniformly hyperreflective from fibrosis and the increased collagen content^18^. Furthermore, as lesions become less vascular and more fibrous, they become more hyperreflective on OCT^19^. Despite this theoretical understanding, it is incredibly difficult to discriminate the two types of tissue on OCT in practice. As a result, in the literature, such PEDs are referred to as fibrovascular PEDs, a mix of fibrous and neovascular tissue^4,13^. To discriminate between the two, we used preprocessing steps to reduce noise and applied a standard deviation filter to characterize local heterogeneity. We then subtracted a factor of this value from the raw intensity. Therefore, pixels with greater local heterogeneity and lower pixel intensity were more likely to be classified as neovascular, aligning with the theoretical understanding of the tissue.

While evaluating different areas within PED, we noticed that serous fluid had the greatest absolute area, while neovascular tissue had the greatest relative percentage. Serous fluid was found in many of the very large PEDs whereas neovascular tissue was found in both large and small PEDs. The mean PED area greatly varied between scans, as did the mean areas of each tissue type. Despite this, repeatability and reproducibility remained greater than 0.80 for all measurements except serous and neovascular ICC for repeatability for grader 2.

Although neovascular and fibrous tissue have similar appearances on OCT, they have different prognostic value. Neovascular tissue is prone to leaking fluid into the PED and surrounding subretinal space. Fibrous tissue, in the form of increased collagen content and reflectivity on OCT, is a sign of response to treatment during anti-VEGF therapy. Fibrovascular PEDs therefore encompass a range of lesions, which can be described by multiple subtypes depending on the presence and amount of neovascular and fibrous tissue. These subtypes have been shown to lead to different patterns of CNV^20^. As a result, knowing the presence and relative size of the type of material within a PED is critical in evaluating disease progression and response to treatment on routine OCT examination of nAMD patients. In our dataset, 17 of the eyes did not have a serous component, of which 13 had both a neovascular and fibrous components. Therefore, quantifying the relative area of fibrous tissue from neovascular tissue provides an additional level of analysis that could not be derived from describing the region as fibrovascular. Furthermore, easy-to-understand metrics estimating the degree of scarring, PEDCI-F, and amount of serous fluid, PEDCI-S, can aid clinicians in directly comparing PEDs during follow up visits while on anti-VEGF therapy. As shown in Fig. 6, the change in composition of the PED was captured by PEDCI metrics and may help quantify treatment response.

The proposed methodology aims to classify serous, neovascular, and fibrous tissue within a PED to estimate the composition of PED. This task can be represented as a semantic segmentation problem within the region of interest, namely the PED. In recent years, the rise of artificial intelligence (AI), specifically deep learning, has shown great promise in accurately completing these tasks^21^. Convolutional neural networks (CNNs) have already been trained to segment lesions such as intraretinal fluid, hyperreflective foci, and drusen^22–24^. However, these methods require large datasets and ground truth labels for training. Unsupervised AI algorithms, as seen in Fig. 5c, do not require ground truth labels for training but are inferior to the more popular supervised AI algorithms^25^. Furthermore, they have increased computational cost compared to traditional image processing. As the accuracy of unsupervised learning algorithms improves, the aforementioned methodology should be revisited. OCT angiography (OCTA) has been postulated to provide vascularity information within a PED, however lack of external validation makes OCTA a poor substitute for ground truth.

A primary limitation of our study was the effect of noise on our analysis. One of the key characteristics we attempted to quantify was local heterogeneity through the local standard deviation of a pixel. However, the nature of OCT imaging introduces signal noise that can be characterized by this same heterogeneity. Normally to correct for such noise, an averaging filter can be utilized, but this would also decrease our ability to capture the desired characteristic. Therefore, averaging filters were only applied after determining the local standard deviation.

Other limiting factors include the sample size and resolution of SD-OCT. As a result of strict inclusion criteria, the sample size used to develop the algorithm was small. Additional studies with external validation are planned. The technology for OCT has vastly improved over the last decade, but clinically available high-resolution SD-OCT can only capture at 4 and 7 µm per pixel in the axial and transverse direction respectively. The average diameter of a capillary is just around 2.5 to 10 µm, which would be only a few pixels in size on an SD-OCT^26^. Therefore, the high-resolution scans used in this study may have had difficulty capturing the dense capillary network of neovascular PEDs. Further studies with higher resolution SD-OCT scans need to be performed to uncover the potential of image processing in PED tissue classification and utility of PEDCI biomarkers.

Other future directions include automated segmentation, volumetric analysis, clinical correlation, and temporal analysis. Automated segmentation of the PED is necessary to limit human error and decrease the time intensive task of manual segmentation. Over the past decade, researchers have been able to segment different lesions within the retina, including PEDs, using CNNs. We plan to integrate CNNs to automate this pipeline. Further investigation must also be conducted to assess the PEDCI within a volumetric space. 3D OCT scans can provide spatial data and may better capture heterogeneity within tissues and fluid. Volumes calculated from such scans will provide more representative relative sizes of serous, neovascular, and fibrous tissue.

In our preliminary analysis, we showed an example of how PEDCI could be used to evaluate changes in PED composition over the course of treatment. Shifts in PED compartments were observed in the holdout patient on follow-up visit, notably an increase in scarring and a reduction in serous fluid. These shifts demonstrated the utility of PEDCI in capturing treatment related changes in a PED for which the total area remained relatively consistent. An increase in PEDCI-F and decrease in PEDCI-S reflect an increase and decrease of fibrous and serous components respectively, which are accompanied by an improvement in visual acuity. While an additional study is underway to assess the clinical correlations of PEDCI over time, these initial findings show promise in the utility of the biomarker. This will allow us to confirm our hypothesis and determine how compartments within the PED shift due to treatment and disease progression.

In conclusion, our study reports an automated image processing approach to estimate the relative sizes of serous, neovascular, and fibrous tissue which showed high agreement between expert clinicians. We also show how these classifications can be used to calculate an easy to digest PEDCI metrics. Knowledge about the composition of a PED can help inform clinicians of the best course of treatment. We hope to conduct future studies on how these areas and PEDCI change with treatment and disease progression.

## Data Availability

The data generated and analyzed in the current study are not publicly available due to protection of patient privacy and are available upon reasonable request from the corresponding author.

## References

[1] Mitchell P, Liew G, Gopinath B, Wong TY (2018). Age-related macular degeneration. The Lancet.

[2] Wong WL (2014). Global prevalence of age-related macular degeneration and disease burden projection for 2020 and 2040: a systematic review and meta-analysis. Lancet Glob. Health.

[3] Ambati J, Ambati BK, Yoo SH, Ianchulev S, Adamis AP (2003). Age-related macular degeneration: Etiology, pathogenesis, and therapeutic strategies. Surv. Ophthalmol..

[4] Chhablani, J. & Ruiz-Medrano, J. *Choroidal Disorders*. (Elsevier/AP, Academic Press is an imprint of Elsevier, 2017).

[5] Spaide RF (2020). Consensus nomenclature for reporting neovascular age-related macular degeneration data. Ophthalmology.

[6] Rastoin O, Pagès G, Dufies M (2020). Experimental models in neovascular age related macular degeneration. International Journal of Molecular Sciences.

[7] Jaffe GJ (2019). Macular morphology and visual acuity in year five of the comparison of age-related macular degeneration treatments trials. Ophthalmology.

[8] Cunningham ET, Feiner L, Chung C, Tuomi L, Ehrlich JS (2011). Incidence of retinal pigment epithelial tears after intravitreal ranibizumab injection for neovascular age-related macular degeneration. Ophthalmology.

[9] Wu PC, Chen YJ, Kuo HK (2011). Retinal pigment epithelial tear after intravitreous triamcinolone acetonide injection for fibrovascular pigment epithelial detachment. Chang Gung Med. J..

[10] Spaide RF (2009). Enhanced depth imaging optical coherence tomography of retinal pigment epithelial detachment in age-related macular degeneration. Am. J. Ophthalmol..

[11] Mrejen S, Spaide RF (2013). Optical coherence tomography: Imaging of the choroid and beyond. Surv. Ophthalmol..

[12] Khanani AM, Eichenbaum D, Schlottmann PG, Tuomi L, Sarraf D (2018). Optimal management of pigment epithelial detachments in eyes with neovascular age-related macular degeneration. Retina.

[13] Hoerster R, Muether PS, Sitnilska V, Kirchhof B, Fauser S (2014). Fibrovascular pigment epithelial detachment is a risk factor for long-term visual decay in neovascular age-related macular degeneretion. Retina.

[14] Shah M (2011). Evaluating intensity normalization on MRIs of human brain with multiple sclerosis. Med. Image Anal..

[15] Vupparaboina KK (2018). Quantitative shadow compensated optical coherence tomography of choroidal vasculature. Sci. Rep..

[16] Girard MJA, Strouthidis NG, Ethier CR, Mari JM (2011). Shadow removal and contrast enhancement in optical coherence tomography images of the human optic nerve head. Investig. Opthalmol. Vis. Sci..

[17] Otsu N (1979). A threshold selection method from gray-level histograms. IEEE Trans. Syst. Man Cybern..

[18] Phillips KG (2011). Dermal reflectivity determined by optical coherence tomography is an indicator of epidermal hyperplasia and dermal edema within inflamed skin. J. Biomed. Opt..

[19] Keane PA (2012). Evaluation of age-related macular degeneration with optical coherence tomography. Surv. Ophthalmol..

[20] Souied EH (2020). Spectral-domain optical coherence tomography analysis of fibrotic lesions in neovascular age-related macular degeneration. Am. J. Ophthalmol..

[21] Schmidt-Erfurth U, Sadeghipour A, Gerendas BS, Waldstein SM, Bogunović H (2018). Artificial intelligence in retina. Prog. Retin. Eye Res..

[22] Gorgi Zadeh, S. et al. *Deep Learning in Medical Image Analysis and Multimodal Learning for Clinical Decision Support Lecture Notes in Computer Science* Ch. Chapter 8, 65–73 (2017).

[23] Schlegl, T. et al. *Fully Automated Segmentation of Hyperreflective Foci in Optical Coherence Tomography Images* (2018).

[24] Venhuizen FG (2018). Deep learning approach for the detection and quantification of intraretinal cystoid fluid in multivendor optical coherence tomography. Biomed. Opt. Express.

[25] Gao S (2022). Large-scale unsupervised semantic segmentation. IEEE Trans. Pattern Anal. Mach. Intell..

[26] Snodderly DM, Weinhaus RS, Choi JC (1992). Neural-vascular relationships in central retina of macaque monkeys (*Macaca fascicularis*). J. Neurosci..

